# Peptide-Functionalized and Drug-Loaded Tomato Bushy Stunt Virus Nanoparticles Counteract Tumor Growth in a Mouse Model of Shh-Dependent Medulloblastoma

**DOI:** 10.3390/ijms24108911

**Published:** 2023-05-17

**Authors:** Luca Marchetti, Flavia Novelli, Barbara Tanno, Simona Leonardi, Veronica Mohamed Hizam, Caterina Arcangeli, Luca Santi, Selene Baschieri, Chiara Lico, Mariateresa Mancuso

**Affiliations:** 1 Laboratory of Biomedical Technologies, Italian National Agency for New Technologies, Energy and Sustainable Economic Development, ENEA, Casaccia Research Center, Via Anguillarese 301, 00123 Rome, Italy; luca.march46@gmail.com (L.M.); flavia.novelli@enea.it (F.N.); barbara.tanno@enea.it (B.T.); simona.leonardi@enea.it (S.L.); veronicamohamedhizam@gmail.com (V.M.H.); 2 Department of Agriculture and Forest Sciences (DAFNE), University of Tuscia, Via S. Camillo De Lellis, 01100 Viterbo, Italy; luca.santi@unitus.it; 3 Laboratory of Health and Environment, Italian National Agency for New Technologies, Energy and Sustainable Economic Development, ENEA, Casaccia Research Center, Via Anguillarese 301, 00123 Rome, Italy; caterina.arcangeli@enea.it; 4 Laboratory of Biotechnologies, Italian National Agency for New Technologies, Energy and Sustainable Economic Development, ENEA, Casaccia Research Center, Via Anguillarese 301, 00123 Rome, Italy; selene.baschieri@enea.it

**Keywords:** TBSV NPs, drug delivery system, doxorubicin, preclinical study, *Ptch1*^+/−^ mice, MB therapy

## Abstract

Sonic hedgehog medulloblastoma (SHH-MB) accounts for 25–30% of all MBs, and conventional therapy results in severe long-term side effects. New targeted therapeutic approaches are urgently needed, drawing also on the fields of nanoparticles (NPs). Among these, plant viruses are very promising, and we previously demonstrated that tomato bushy stunt virus (TBSV), functionalized on the surface with CooP peptide, specifically targets MB cells. Here, we tested the hypothesis that TBSV-CooP can specifically deliver a conventional chemotherapeutic drug (i.e., doxorubicin, DOX) to MB in vivo. To this aim, a preclinical study was designed to verify, by histological and molecular methods, if multiple doses of DOX-TBSV-CooP were able to inhibit tumor progression of MB pre-neoplastic lesions, and if a single dose was able to modulate pro-apoptotic/anti-proliferative molecular signaling in full-blown MBs. Our results demonstrate that when DOX is encapsulated in TBSV-CooP, its effects on cell proliferation and cell death are similar to those obtained with a five-fold higher dose of non-encapsulated DOX, both in early and late MB stages. In conclusion, these results confirm that CooP-functionalized TBSV NPs are efficient carriers for the targeted delivery of therapeutics to brain tumors.

## 1. Introduction

The signaling deregulation of sonic hedgehog (SHH) is one of the molecular signatures of medulloblastoma (MB), the most common malignant pediatric brain tumor. This cancer molecular variant originates from granule cell precursors (GCPs), the cellular population forming the external granule cell layer (EGL) during cerebellar development [1]. SHH secreted from Purkinje cells is the most efficacious mitogen for GCPs expansion [2]. SHH-MBs are hypothesized to be extremely heterogeneous due to the bimodal distribution in the human population, characterized by peaks in infants under 3 years of age and adults over 16 years of age and only a small onset in childhood (4–15 years). Integrative genomic approaches have recently demonstrated that pediatric and adult SHH-MBs are clinically, transcriptionally, genetically, and prognostically distinct [3]. Although current standard therapy (i.e., surgical resection, radiation, and chemotherapy) has improved survival to 70–80%, infant MBs (<5 years of age) have a worse outcome as the use of radiation therapy is very limited due to multiple complications that occur in long-term survivors (i.e., a decline in the IQ levels and cognitive function, as well as impairment of hearing, fertility, and physical performance, in addition to the risk of secondary malignancies) [4]. To overcome these limitations, in the last decades, several small molecule inhibitors of the Shh pathway have been considered as promising candidate drugs for SHH-driven tumors [5]. The activation of SHH signaling requires binding of SHH to the twelve-pass transmembrane receptor Patched1 (PTCH1)-mediated Smoothened (SMO) receptor complex (PTCH1-SMO) and induction of a downstream signaling cascade [6]. Based on the high frequency of *PTCH1* mutations in SHH-MBs, especially SMO inhibitors have been tested in clinical trials; however, their therapeutic efficacy is transitory due to the resistance acquisition conferred by mutations occurring in *SMO* or in its downstream genes [2].

Along with the development of new drugs, nanotechnological-based approaches represent a new challenge to improve clinical outcomes, especially for pediatric cancers. Nanoparticle (NP)-based drug delivery systems offer several advantages, decreasing the toxicity of the therapy on non-target organs providing a sustained or controlled drug release, improving the pharmacokinetic properties of the loaded compounds, and achieving a targeted drug delivery by surface functionalization [7]. Among many NP-mediated drug delivery systems developed for the treatment of SHH-MB [8,9,10], we previously tested the use of an engineered plant virus [11]. Tomato bushy stunt virus, the type-member of the genus *Tombusvirus*, is characterized by an icosahedral capsid of ~32 nm in diameter composed of 180 subunits of a single type of coat protein (CP). TBSV NPs are neither toxic nor teratogenic and do not induce alterations of tissues/organs when administered intravenously [12,13]. TBSV has been functionalized on the outer surface with CooP peptide (a 9 aa peptide, sequence: CGLSGLGVA), obtaining TBSV-CooP NPs. The CooP peptide has been selected by the in vivo screening of a phage display library and classified as a glioblastoma homing peptide, with the mammary-derived growth inhibitor (MDGI/FABP3) identified as its interacting partner [14,15]. TBSV-CooP, when loaded into the inner cavity with doxorubicin (DOX), has been proven to induce a mortality rate of primary murine MB cells of 90% with a DOX dose five-fold lower (5 μM) than free drug (25 μM). Furthermore, when tested in vivo, TBSV-CooP was demonstrated to have a higher ability to target MB cells compared to TBSV-WT [11].

Here, we took advantage of a well-characterized mouse model of Shh-dependent MB, the *Patched1* heterozygous (*Ptch1*^+/−^) mice, to test the therapeutic efficacy of the TBSV-NP-based delivery system. *Ptch1*^+/−^ mice, maintained on CD1 genetic background, spontaneously develop a low percentage of MBs by 20 weeks of age, but the process can be strongly promoted by neonatal irradiation [16]. Furthermore, preneoplastic MB stages (i.e., hyperplastic cerebellar lesions of increasing degree with altered cellular morphology and size) are present in the cerebellum of asymptomatic mice at high incidence [17,18]. The peculiarity of the adopted animal model has allowed us to establish that a single dose of DOX-loaded TBSV-CooP was able to induce DNA damage followed by the activation of pro-apoptotic pathways in full-blown MBs, while multiple doses were able to inhibit tumor progression of early MB stages, with also an important absence of undesirable side effects. These results corroborate the use of this plant-virus-based delivery platform as a novel therapeutic strategy against MB.

## 2. Results

### 2.1. TBSV-CooP NP Production and Drug Loading

TBSV NPs were genetically engineered to expose the CooP peptide on the virus surface, fused to the C-terminus of each CP subunit. The chimeric TBSV NPs are stable through several reinfection cycles and maintain the correct structure and monodispersion state also after the drug loading protocol, which contemplates capsid swelling, drug infusion, and re-association procedure [11]. Here a batch of purified TBSV-CooP NPs sufficient for the entire in vivo experiment has been produced on a large scale in *N. benthamiana* plants. TBSV NPs have been purified and quantified, obtaining a yield of about 1 mg of virus/g of fresh leaves, consistent with previous data. The preparation purity has been verified by Coomassie Blue staining of SDS-PAGE, confirming the unique presence of the viral CP (both as a monomer and as a dimer/aggregate). TBSV-CooP NPs have been then loaded with DOX, resulting in a preparation in which each virion was carrying around 1898 DOX molecules, i.e., 123 ng of DOX/μg of virus (corresponding to a loading capacity of 12.5% and an encapsulation efficiency of 38%).

### 2.2. DOX-Loaded TBSV-CooP NPs Inhibit Progression of MB Pre-Neoplastic Lesions

At 6 weeks of age, almost the totality of neonatally-irradiated *Ptch1*^+/−^ mice are characterized by the presence of microscopically recognizable cerebellar abnormalities, which progress into full-blown tumors [18]. Thus, to evaluate the capability of the TBSV-CooP drug delivery strategy to efficiently reach the target, inducing a therapeutic effect, we designed a preclinical study focused on the early phases of MB development. To this aim, we compared the effect induced by a dose of free DOX (5 mg/kg) with a five-fold lower dose (1 mg/kg) entrapped in TBSV-Coop NPs. Clinically, DOX is administered intravenously at doses of 1–10 mg/kg into the tail vein. If 1–10% of the injected dose reaches the tumor site, then the resulting intratumoral dose would equate to 0.01–1 mg/kg [19]; thus, our dose is within a clinically relevant use range of DOX.

**Figure 1:**
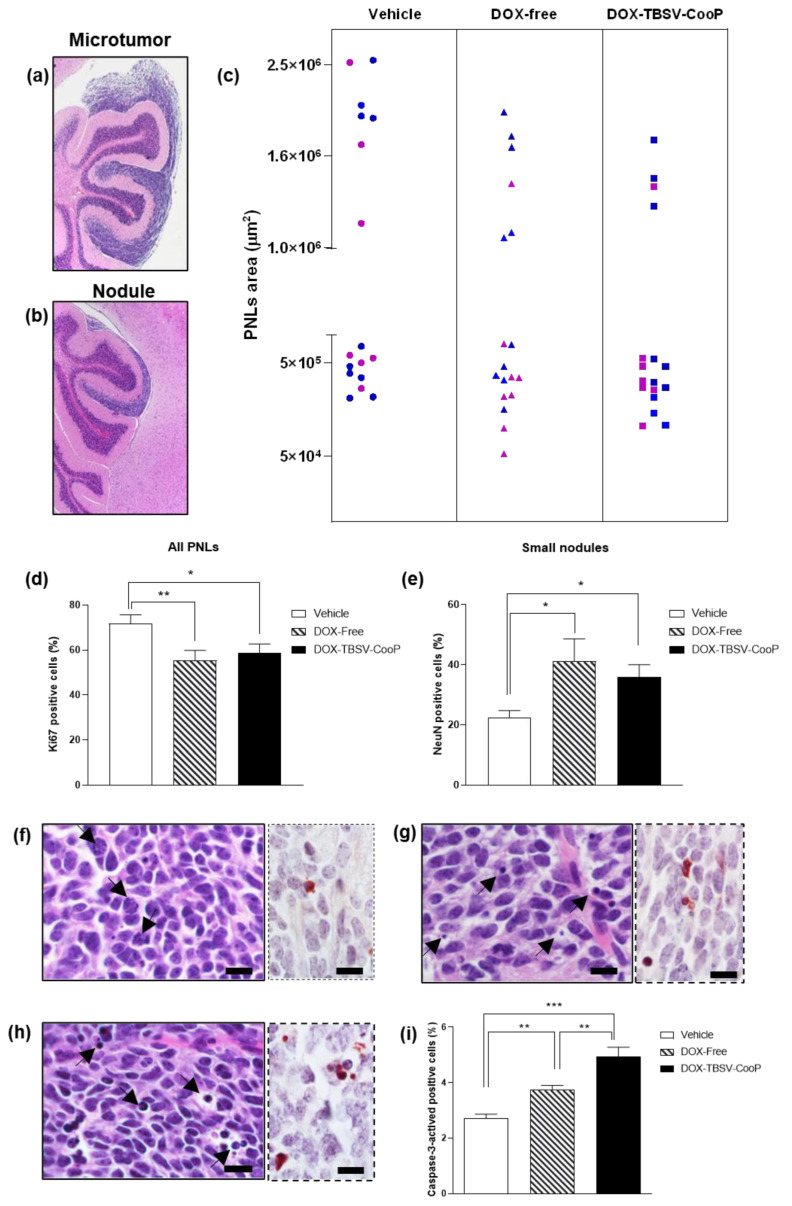
Histological and immunohistochemical analyses of MB PNLs. (**a**). Representative image of a PNL classified as microtumor (area > 1 × 10^6^ μm^2^). (**b**) Representative image of a PNL classified as small nodule (area < 1 × 10^6^ μm^2^). (**c**) Distribution of PNLs according to their cross-sectional area in sham (*n* = 17), DOX-free (*n* = 18), and DOX-TBSV-CooP (*n* = 17) groups. Blue = male mice; magenta = female mice. (**d**) PNLs proliferation index calculated as percentage of Ki67-positive/total cells. Datasets represent the mean ± SEM. * *p* < 0.05; ** *p* < 0.01 (Student’s *t*-test). (**e**) Rate of cell differentiation in small nodules calculated as percentage of NeuN-positive/total cells. Datasets represent the mean ± SEM. * *p* < 0.05 (Student’s *t*-test). Representative images of PNL sections from (**f**) sham, (**g**) DOX-free, and (**h**) DOX-TBSV-CooP groups. Arrowhead in (**f**) = phases of mitosis; arrowhead in (**g**) and (**h**) = apoptosis. Inserts in (**f**–**h**) = immunostaining with an antibody against caspase-3-activated. (**i**) Apoptotic index calculated as percentage of caspase-3-positive/total cells. Datasets represent the mean ± SEM. ** *p* < 0.01; *** *p* < 0.001 (Student’s *t*-test). Scale bars = 10 μm.

From 6 to 8 weeks of age, mice were treated 4 times (2 times per week) with DOX-free, DOX-TBSV-CooP, or vehicle (sham group) and sacrificed 3 days after the last treatment. According to histological and dimensional analyses, pre-neoplastic lesions (PNLs) found in all analyzed FFPE mouse brains were classified as asymptomatic microtumors (area > 1 × 10^6^ μm^2^; Figure 1a) or small nodules (area < 1 × 10^6^ μm^2^; Figure 1b). Although the total incidence of positive mice with cerebellar abnormalities was 100% for each experimental group, a different size distribution of PNLs was observed among the groups. As shown in Figure 1c, in the brains of vehicle-treated mice, 41% (7/17) of microtumors and 59% (10/17) of small nodules were found, with a global proliferation index of 72% (ki67-positive/total cells; Figure 1d). The frequency of microtumors decreased to 33% (6/18) and 23.5% (4/17) in DOX-free and DOX-TBSV-CooP-treated groups, respectively. The corresponding increased frequency of less progressed PNLs (67% and 76.5%) suggests a slowing down of tumor progression (Figure 1c,d) in both treated groups. This conclusion is corroborated by the statistically significant inhibition of the proliferation index in DOX-free (55.3%; *p* = 0.0097) and DOX-TBSV-CooP (58.7%; *p* = 0.0247) PNLs, compared to that quantified in PNLs from the vehicle-treated group. Representative histological images of MB PNLs immunostained with Ki67 antibody are shown in Figure S1.

Furthermore, immunostaining of small nodules with an antibody directed against NeuN, a marker of neuronal differentiation, highlights a statistically significant higher percentage of NeuN-positive cells in DOX-free (41.1%; *p* = 0.0241) and in DOX-TBSV-CooP (35.9%; *p* = 0.0117) treated animals compared to the sham (22.5%) group, indicating that DOX induces the regression of small nodules to normal tissue, thus decreasing propensity to develop into MB (Figure 1e and Figure S1). Notably, in PNLs from DOX-free and DOX-TBSV-CooP-treated groups, several figures associated with irreversible cell injury (apoptosis) were morphologically recognized (Figure 1g,h), also confirmed by immunostaining with an anti-caspase-3 antibody compared to only sporadic apoptotic cells in vehicle-treated counterparts, where many cells in mitosis are present (Figure 1f–h, inserts). Quantification of apoptotic cells clearly demonstrated a statistically significant increase in the apoptotic rate in MB from DOX-free and DOX-TBSV-CooP-treated groups (Figure 1i); notably, MB treated with DOX-TBSV-Coop showed the highest level of damaged cells (*p* < 0.0001 vs. vehicle; *p* = 0.0040 vs. DOX-free).

**Figure 2:**
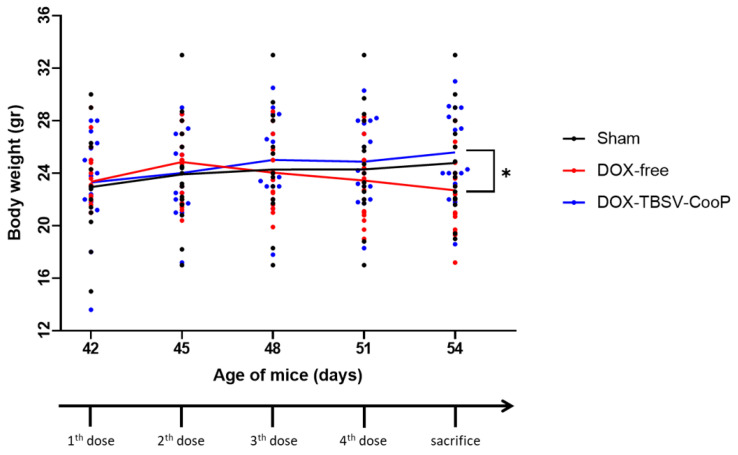
Evaluation of body weight registered immediately before each i.v. injection of NaAc (vehicle), DOX-free, and DOX-TBSV-CooP, and at the time of organ collection (54 days). Lines represent the mean weight of each experimental group. Dots represent individual mice. * *p* < 0.05 (Student’s *t*-test).

Although microscopic examination of organs (spleen, kidneys, lung, and liver) collected from mice in all the experimental groups did not show evident alterations of normal structure nor the presence of cell injury (Figure S2), mice treated with DOX-free manifested the symptoms of possible systemic toxicity as demonstrated by body weight loss after the third injection that was statistically significant compared to the vehicle-treated group after the last treatment (*p* = 0.0429; Figure 2). On the contrary, no variation in body weight was observed in DOX-TBSV-CooP-treated mice all along the duration of the experiment.

Overall, these results clearly demonstrate the advantage offered by the TBSV-based drug delivery system in contrasting MB PNLs towards their evolution in overt tumors. Our TBSV platform is able to inhibit tumor cell proliferation and promote cell death as well as cell differentiation, despite using a drug dose that was five times lower, without any observable undesirable side effects.

### 2.3. Effects of DOX-Loaded TBSV-CooP NPs on Full-Blown Tumors

**Figure 3:**
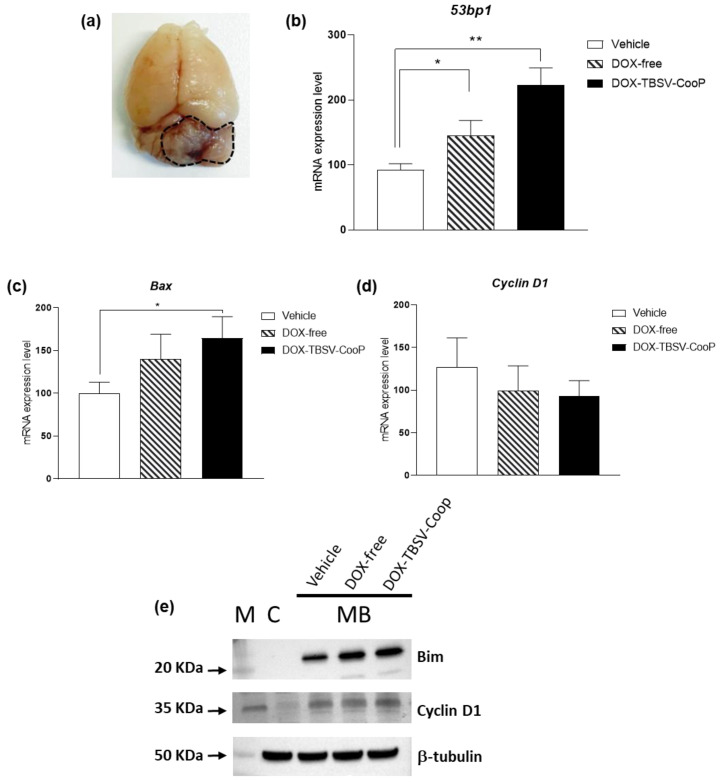
Effects induced by DOX-loaded TBSV-CooP NPs on overt MB. (**a**) Representative image of an explanted brain from a symptomatic *Ptch1*^+/−^ mouse; dashed line = tumor mass. Evaluation of (**b**) *53pb1*, (**c**) *Bax*, and (**d**) *Cyclin D1* expression levels, determined by qPCR analysis after a single i.v. injection of vehicle, DOX-free, and DOX-loaded TBSV-CooP in mice with clear MB symptoms. Each dataset represents the mean ± SEM of three independent biological replicates. * *p* < 0.05; ** *p* < 0.01 (Mann–Whitney test). (**e**) Western blot analysis of Bim and Cyclin D1 expression. β-tubulin was used to normalize protein loading. C = normal cerebellum. The marker (M) for Bim and β-tubulin is Precision Plus Protein Standard Dual Color (BIO-RAD), while for Cyclin D1, it is SharpMass VI protein standard (EuroClone). Densitometry of each band is shown in Supplementary Table S2.

*Ptch1*^+/−^ mice, irradiated at post-natal day 2 with a single dose of 3 Gy of X-rays, develop full-blown MBs starting from 10 weeks post-irradiation with maximum tumor incidence (60%) reached by 20 weeks [18]. Tumor development is accompanied by clearly recognizable symptoms (i.e., weight loss, skull deformation, impaired balance, and paralysis), and since the onset of these symptoms, mice survival is reduced to a few days. At this stage of MB development, tumor mass is well-recognizable in the posterior cranial fossa, and it is easily detached from the remaining normal cerebellum (Figure 3a). To establish if our platform of drug delivery could also offer a therapeutic benefit at this late stage of MB development, symptomatic mice were i.v. injected into the tail vein with vehicle (*n* = 5), DOX-free (*n* = 5), or DOX-TBSV-CooP (*n* = 5), and 24 h later, tumors were collected for RNA and protein extractions.

By qPCR, mRNA expression levels of genes involved in DNA repair, apoptosis, and cell proliferation were initially assessed. As shown in Figure 3b, a statistically significant higher expression of the 53pb1 gene, a classic late marker of DNA damage response, was observed in both treated groups compared to the sham group. To note, in MBs from mice treated with DOX-free, 53pb1 mRNA expression was 1.26-fold higher with respect to controls (*p* = 0.0480); on the contrary, when DOX was delivered through the engineered virus, its capability to induce double strain breaks was more effective despite the five times lower concentration (two-fold increase vs. vehicle, *p* = 0.0025; 1.56-fold increase vs. DOX-free, *p* = 0.0728). In support of these data, the expression levels of the pro-apoptotic gene Bax were also significantly up-regulated in tumors from DOX-TBSV-CooP compared to vehicle-treated mice (*p* = 0.0480; Figure 3c). Analyzing Cyclin D1, an important regulator of cell cycle progression, we did not find significant variation in the expression levels among groups (Figure 3d); however, a decreasing trend in both treated groups is consistent with the lower proliferation index of PNLs MB (Figure 1d), suggesting that repeated administrations of the DOX-carrying NPs would be able to inhibit tumor cell proliferation also during the late stage of MB development. As shown in Figure 3e, Western blot analyses of Bim, a direct activator of Bax [20], and Cyclin D1 are in agreement with the mRNA expression levels results.

**Figure 4:**
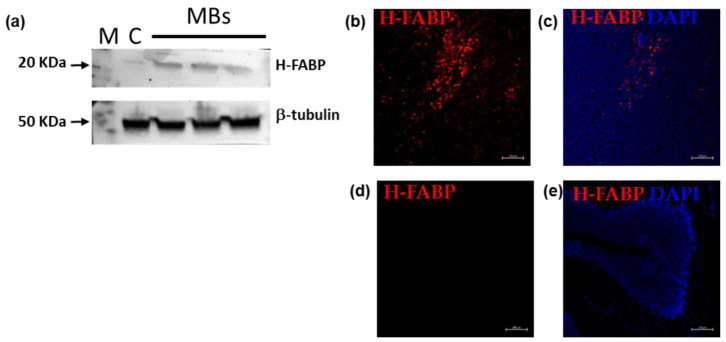
Evaluation of H-FABP expression in MB. (**a**) Western blot analysis of H-FABP expression in three different spontaneous MBs from untreated mice. β-tubulin was used to normalize protein loading. Cryosections of MB (**b**,**c**) and normal cerebellum (**d**,**e**) stained with anti-H-FABP antibody (red); cell nuclei stained with DAPI (blue). C = normal cerebellum. M = SharpMass VI protein standard (EuroClone). Densitometry of each band is shown in Supplementary Table S2. Scale bars = 100 μm.

The primary interacting partner of the CooP peptide was identified to be the mammary-derived growth inhibitor (MDGI/FABP3) [15]. By molecular dynamic approach, we previously demonstrated that the binding mode of CooP with FABP3 was more stable when CooP was fused to the viral coat protein, suggesting the hypothesis that the internalization by MB cells could be receptor-mediated [11]. Western blot and immunofluorescence analysis of H-FABP expression in three independent MBs clearly showed an increase in protein levels in tumors compared to a normal cerebellum (Figure 4a,b). These findings highlight that the higher induction of DNA damage and apoptosis obtained by DOX-TBSV-CooP can be attributed, at least for Shh-dependent MB, to the presence of FABP3 and its internalization activity.

### 2.4. Antibody Titration

The antibody response against the virion shell composed of a single type of CP has been evaluated. The dense experimental schedule of NP administrations concentrated over 2 weeks did not allow the sampling of the sera after every single dose; consequently, the analysis was performed by ELISA at the end of the experiment, evaluating individual CP-specific IgG titers. No anti-TBSV antibodies have been detected in the sera of the vehicle-treated mice, while CP-specific IgGs were found in DOX-TBSV-CooP-treated mice with endpoint titers spanning from 1:35,841 to 1:66,805 (average 1:48,128), and no statistical differences between male and female.

## 3. Discussion

Conventional cancer treatment consists of delivering the chemotherapy drugs systemically through the circulatory system, and this often results in whole-body dispersion and premature release before tumors are reached. Consequently, the dose of the drug must be increased considerably in order to reach the target organ in an adequate quantity to be effective; however, this strategy has the disadvantage of causing undesired damage to normal tissues or cells. Hence, one of the major goals of precision cancer medicine development is to improve the efficacy of the delivered drug by improving its targeting. NPs may have great clinical potential in this field. Viral NPs, and plant virus NPs in particular, are intriguing from a nanomaterial science standpoint, given their self-assembling architecture and the easiness of production that can reach milligrams in a lab-scale frame. The intrinsic quality-control mechanism ensures that all the particles are almost similar in size and shape, a feature, among several others, that is impossible to obtain with synthetic nanomaterials, achieving on the whole optimal cargo loading capacity, targeting efficiency, biocompatibility, ease of manufacturability, and cost-effectiveness. Finally, plant virus NPs can be custom modified by genetic engineering and are biodegradable, a clear advantage over low modification efficiency typical of synthetic materials, which rely on chemical synthesis and over their inherent persistence in the body for extended periods.

In this context, a TBSV-based drug system of targeted delivery for Shh-dependent MB treatment has been developed and characterized, moving from preliminary in vitro data to in vivo efficacy experiments. CooP-functionalized and DOX-loaded TBSV NPs have been demonstrated to be specifically uptaken by primary cultures of Shh-MB cells, allowing a five-fold reduction in the effective drug dose to induce a 90% decrease in tumor cells viability [11]. On the basis of in silico simulations, it was hypothesized that DOX-TBSV-CooP NP internalization by MB cells could be mediated by MDGI/FABP3, the receptor expressed on glioblastoma cells recognized by CooP [14]. Here, with different experimental approaches, it was found that H-FABP, the best-characterized member of the FABP family encoded by the FABP3 gene [21], is also expressed in Shh-MBs. The specific receptor-mediated mechanism of uptake by target cells explains well why TBSV NPs can achieve a therapeutic effect against MB growth with a lower dose of DOX than free-drug.

The current chemotherapy approach adopted for MB treatment does not include DOX [22]. Nevertheless, DOX was selected as the drug to be delivered through TBSV NPs because it is fluorescent, thus allowing proper optimization of an ad hoc particle loading protocol. In agreement with the well-known capability of DOX to activate different regulatory mechanisms inducing either apoptosis or cell death [23], in both treated experimental groups at early and also late stages of MB development, we found a significant increase in apoptosis and activation of pro-apoptotic pathways. Notably, when DOX was delivered through the engineered TBSV NPs, its capability to induce apoptosis was significantly higher than when freely administered at a five-fold higher concentration. The higher pro-apoptotic effect induced by DOX-TBSV-CooP correlates with the significantly higher expression of the DNA damage response factor *53bp1*, found in the tumor already after a single injection of TBSV NPs. Although a single dose of engineered TBSV NPs was unable to significantly change *cyclin D1* expression levels, the reduced content in Ki67 resulting from the analysis of early stages of MB development suggests that TBSV-based DOX delivery platform inhibits tumor cell proliferation as effectively as a five-fold higher dose of free-DOX. Another important advantage offered by the administration of the drug through the proposed delivery system is the absence of systemic toxic effects. Although no cytotoxic damage to non-target organs was found in both treated groups, animals treated with multiple doses of free-DOX suffered from a significant loss of body weight. This effect may be related to the decrease in fat and skeletal muscle mass caused by DOX due to its inhibitory effects on AMPk, resulting in hyperglycemia and insulin resistance and culminating in atrophy, weight loss, and anorexia [24]. Since TBSV is a “protein-based cage”, a potential disadvantage of its use as a drug delivery system is that it is, per se, immunogenic. This is the reason why the antibody response against the viral CP was evaluated. The titers of the antibodies after repeated administrations were higher in comparison to those measured after a single inoculum [11]. Immunogenicity is a translational challenge as the production of carrier-specific antibodies has also been reported for various inorganic and synthetic systems; for example, iron oxide NPs, liposomes, multi-walled carbon nanotubes [25,26,27], and obviously other plant viruses [28]. Moreover, the immune response is one of the natural clearance mechanisms, and it does not pose per se any potential health risk or adverse effect. Generally, it is assumed that the immunogenic properties of protein-based carriers can be attenuated by stealth polymer coating, as polyethylene glycol (PEG) [29] is able to decrease serum protein adsorption, thus clearance by the mononuclear phagocyte system. Nevertheless, more recently, this paradigm has been rapidly changing, and antibody production against PEG in humans has also been reported [30,31,32]. Even if unintended immune responses may result in unfavorable accelerated clearance of delivery systems, it apparently does not dramatically affect the efficacy of the TBSV-based delivery strategy.

## 4. Materials and Methods

### 4.1. Production of DOX-Loaded TBSV-CooP NPs

The sap obtained by homogenizing the leaves of an infected *Nicotiana benthamiana* plant in 1× Phosphate Buffered Saline (PBS: 151 mM NaCl, 8.4 mM Na_2_HPO_4_ × 12H_2_O, 1.86 mM NaH_2_PO_4_ × H_2_O, pH 7.2) was used to infect on larger scale six to eight weeks old *N. benthamiana* plants, grown under standard controlled conditions (16/8 h day/night cycle, 25 °C, 65% humidity, daily light integral 3.9 moles/day, photosynthetically active radiation 136 μmol/m^2^/s) in a containment greenhouse by abrading the adaxial side of 2 leaves/plant with carborundum (silicon carbide; VWR International, Radnor, PA, USA). TBSV-CooP NPs were produced on a large scale in plants, as already described [11]. 

Briefly, TBSV-CooP NPs were purified from powdered leaves; after homogenization and centrifugation, the supernatant was ultra-centrifuged (1 h at 90,000× *g* at 4 °C), and the pellet resuspended in 50 mM sodium acetate, pH 5.3 [33].

**Figure 5:**
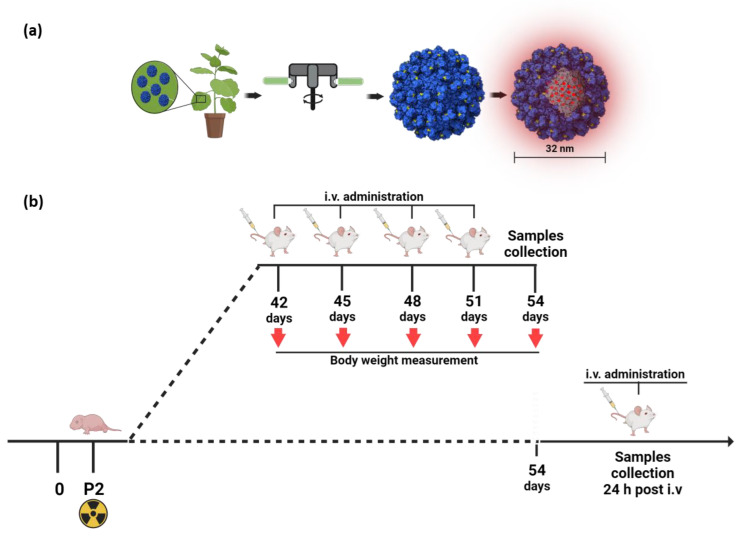
Representative scheme of TBSV NP production (**a**) and experimental administration schedule (**b**).

For DOX loading, the protocol set up in [11] was followed. Briefly, approximately 2 mg/mL of purified TBSV NPs were incubated in swelling buffer (0.1 M Trizma base, 50 mM EDTA, pH 8.0) for 1 h at RT and a 5000 molar excess in DOX (0.324 μg DOX/μg virus) was added overnight at 4 °C. After incubation in association buffer (0.2 M NaAc, 25 mM CaCl_2_, 25 mM MgCl_2_, pH 5.2) at RT for 1 h in agitation, DOX excess was removed by overlaying the preparation onto a 20% sucrose cushion ultracentrifuged at 100,000× *g* for 1 h at 4 °C. Pellets were resuspended overnight in association buffer and stored at 4 °C. The procedure for TBSV NP production is summarized in Figure 5a.

Finally, DOX-loaded TBSV-CooP NPs were analyzed by a UV/VIS plate reader (Glomax, Promega) at 490 nm to determine the number of DOX molecules loaded/virion. A DOX calibration curve was generated, and the concentration of DOX associated with NPs was determined using the Lambert–Beer law [34]. The drug encapsulation efficiency (EE) and loading capacity (LC) of DOX in TBSV NPs were determined according to the following formulae:

EE (%) = amount of DOX in the NPs/total amount of DOX added (×100%)

LC (%) = amount of DOX in the NPs/NPs weight (×100%)

### 4.2. In Vivo Experimental Design

*Ptch1*^+/−^ mice of both sexes were obtained at the ENEA Casaccia (Rome, Italy) animal facility by crossing *Ptch1*^+/−^ heterozygous males with CD1-wild-type females and vice versa and selected after genotyping as previously described [18]. At postnatal day 2 (P2), pups were whole-body irradiated with a single 3 Gy dose of X-rays (dose rate = 0.89 Gy/min) using a Gilardoni CHF 320 G X-ray generator (Gilardoni S.p.A., Mandello del Lario, Italy). To test the inhibition of tumor progression, at six weeks of age, a total of 52 *Ptch1*^+/−^ mice of both sexes (ratio of males:females, 1:1) were randomized into 3 groups and injected intravenously (i.v.) by tail vein 2 times per week for 2 weeks with 150 μL of vehicle (*n* = 17; NaAc pH 5.2), DOX-TBSV-CooP (*n* = 18; DOX concentration 1 μg/g in NaAc), and DOX-free (*n* = 17; 5 μg/g in NaAc). During the treatment, mice were monitored daily to check their general health status and weighed immediately before each treatment; three days after the last injection, mice were weighed, sacrificed, and organs (brain, spleen, kidneys, lung, liver), as well as blood, were collected. For molecular analyses, *Ptch1*^+/−^ mice (*n* = 6 for each experimental group; ratio of males:females, 1:1) with symptomatic MBs were i.v. treated by tail vein with a single dose of vehicle, DOX-TBSV-CooP, and DOX-free, with the same aforementioned concentration, and sacrificed 24 h after the treatment. Tumors were collected in two aliquots and immediately frozen in liquid nitrogen, then stored at −80 °C for RNA and protein extraction. The experimental administration schedule is summarized in Figure 5b. Throughout the experimental duration, animals were housed under conventional conditions with food and water available ad libitum and a 12 h light-dark cycle.

### 4.3. Histology, Morphometry, and Immunohistochemistry

The collected organs were formalin-fixed and paraffin-embedded (FFPE) for routine histology. To assess the percentage of PNLs, the entire cerebellum was examined, recovering FFPE brain sections with intervals of 70 μm. At the recognition of abnormal cerebellar regions—in general, defined as PNLs—sections were collected and stained with hematoxylin and eosin (H&E). PNLs cross-sectional areas were carried out using imaging software NIS-Elements BR 4.00.05 (Nikon Instruments Europe B.V., Campi Bisenzio, Italy).

For immunohistochemistry, PNL-positive FFPE brain sections (4 μm) were dewaxed for 20 min at 56 °C and incubated in citrate buffer pH 6.0 for 20 min at 95 °C. After peroxidases inhibition by 3% H_2_O_2_ for 10 min, sections were incubated with primary antibodies anti-Ki67 (Bethyl, Montgomery, TX, USA), anti-NeuN (Merck Millipore, Darmstadt, Germany), and anti-caspase-3-activated (Cell Signaling Technology, Danvers, MA, USA) for 1 h at room temperature in a moist chamber. After incubation with the secondary anti-rabbit antibody (Bethyl), the antigen–antibody reaction was revealed by DAB (Dako, Agilent Technologies, Santa Clara, CA, USA) and analyzed by HistoQuest (TissueGnostics, Vienna, Austria) software.

### 4.4. RNA Extraction and Real-Time qPCR

Tumor masses (~30 mg each) were homogenized with gentleMACS™ Octo Dissociator (Miltenyi Biotec, Bergisch Gladbach, Germany) and RNA extracted with the RNeasy Mini Kit (QIAGEN, Hilden, Germany). cDNA synthesis was performed using the High Capacity cDNA reverse transcription kit (Applied Biosystems, Foster City, CA, USA), and qPCR was carried out by StepOnePlus™ Real-Time PCR System (Applied Biosystems), using Power SYBR^®^ Green PCR Master Mix (Applied Biosystems). Relative gene expression was quantified using *Glyceraldehyde-3-phosphate* (*Gadph*) as housekeeping gene. Oligonucleotide primers used for qPCR are listed in Table S1. The ΔΔCt quantitative method was used to normalize the expression of the reference gene and to calculate the relative expression level of target genes.

### 4.5. Western Blot

Total proteins were extracted from ~15 mg of previously collected tumor tissues (*n* = 6 for each experimental group) using T-PER™ Tissue Protein Extraction Reagent (Thermo Fisher Scientific, Rodano, Italy) supplemented with proteinase and phosphatase inhibitors following manual procedure and subsequently quantified by Bradford assay. For each experimental group, an equal amount of proteins was pooled together in a total of 50 µg. Then, the samples were mixed with SDS loading buffer and heated for 5 min at 100 °C. Proteins were separated on 4–20% Tris-glycine TGX precast gel (BIO-RAD, Hercules, CA, USA) in reducing and denaturing conditions before transferring onto PVDF membrane by using the Trans-Blot Turbo Transfer System (BIO-RAD). Membranes were blocked with 5% non-fat dry milk in PBS containing 0.5% (*v*/*v*) Tween-20 (PBS-T) at 37 °C for 2 h. After blocking, membranes were incubated overnight at 4 °C with primary antibodies, diluted in 3% non-fat dry milk in PBS-T, against H-FABP (Abcam, Cambridge, UK; 1:500), Bim (Cell Signaling Technology; 1:1000) and 53pb1 (Abcam; 1:1000). Housekeeping anti-β Tubulin (Abcam; 1:2500) was incubated at RT for 45 min. After washing three times with PBS-T, membranes were incubated at RT for 1 h with horseradish peroxidase-conjugated goat anti-rabbit polyclonal antibody (Abcam; 1:5000 for target genes and 1:15,000 for housekeeping) diluted in 3% non-fat dry milk in PBS-T. The signal development was obtained on washed membranes by enhanced chemiluminescence (Amersham Biosciences, Amersham, UK) and the images acquired through iBright Imaging System (Thermo Fisher Scientific).

### 4.6. Immunofluorescence

Symptomatic *Ptch1*^+/−^ (*n* = 3) and wild-type (*n* = 3) mice were perfused via the left ventricle using 20 mL of PBS. Brains were collected and fixed in cold 4% *w*/*v* paraformaldehyde in PBS at 4 °C for 24 h, washed in PBS at RT for 1 h, and cryoprotected in 30% *w*/*v* sucrose at 4 °C overnight. Cryoprotected and fixed tissues were frozen in optimal cutting temperature compound (Killik-O.C.T. Compound; Bio-Optica, Milan, Italy), cryosectioned at 10 μm, and stored at −20 °C. Air-dried tissue sections were rehydrated in PBS for 10 min, permeabilized in PBS + 0.2% *v*/*v* Triton at RT for 10 min thus incubated with anti-H-FABP antibody (Abcam, 1:200) at 4 °C, overnight. After washing, incubation with secondary Alexa Fluor 564 goat anti-rabbit antibody (Abcam; 1:500) was performed at RT for 30 min, followed by washing and nuclear counterstaining with DAPI (1 μg/mL in PBS) at RT for 8 min. Images were analyzed using Zeiss ZEN PRO software (Zeiss, Jena, Germany).

### 4.7. Enzyme-Linked Immunosorbent Assay (ELISA) for Antibodies Titration

Serum samples belonging to individual mice in the DOX-loaded TBSV-CooP-treated group were analyzed by ELISA to determine the concentrations of anti-TBSV CP antibodies. Briefly, ELISA Maxisorp plates (ThermoFisher Scientific, Waltham, MA, USA) were coated overnight with 100 μL 1× Carbonate Buffer (0.2 M Na_2_CO_3_/NaHCO_3_ at pH 9.4) containing 0.5 μg of purified TBSV-WT. After washing three times with 1× PBS supplemented with 0.1% Tween-20, and twice in PBS, plates were blocked in PBS-5% low-fat milk for 2 h at 37 °C. Then, after washing as before, 100 μL of individual mouse serum dilutions in 1× PBS were added in triplicate to wells and incubated overnight at 4 °C. The presence of bound murine IgG antibodies was detected using an HRP-labelled sheep anti-mouse IgG polyclonal antibody (GE Healthcare Europe GmbH, Opfikon, Switzerland; 1:2500), incubated for 1 h at 37 °C and revealed, after washing, with 2,2-azino-bis (3-ethylbenzthiazoline-6-sulphonic acid) (ABTS; SeraCare, Milford, CT, USA) as substrate. The colorimetric reaction was measured at OD_405nm_ using a microplate absorbance reader (Tecan, Mannedorf, Switzerland). Endpoint titers were defined as the reciprocal of the highest serum dilution giving an absorbance ≥ 0.1 OD above the blank (absorbance of the sera of sham-treated mice). Geometric mean titers were determined for each group.

### 4.8. Statistics

Analyses were performed using GraphPad Prism v.7 for Windows (GraphPad Software, San Diego, CA, USA). We used Student’s *t*-test and Mann–Whitney test for comparison between groups. *p* values ≤ 0.05 were considered statistically significant.

## 5. Conclusions

These results demonstrated the great versatility of TBSV NPs as a drug delivery system, which is grounded on a finely tunable plant-based production and on different approaches for functionalization, such as genetic engineering of the structural proteins and cargo loading. By confirming non-toxicity, it was verified that TBSV NPs have great potential, with the ability to specifically convey a chemotherapy drug to tumor cells in a murine model of MB, reducing tumor progression and allowing one to decrease the drug dose. It would be interesting to test the efficacy of the TBSV-based drug delivery platform in other MB or tumor types since this technology seems to encompass all the characteristics of an elective candidate in theranostics applications.

## Data Availability

The data presented in this study are available on request from the corresponding authors.

## Supplementary Materials

The following supporting information can be downloaded at: https://www.mdpi.com/article/10.3390/ijms24108911/s1.
