# Supplementary material for: Peptide-Functionalized and Drug-Loaded Tomato Bushy Stunt Virus Nanoparticles Counteract Tumor Growth in a Mouse Model of Shh-Dependent Medulloblastoma
Source: Int J Mol Sci. 2023 May 17;24(10):8911. doi: 10.3390/ijms24108911 (PMC10219057; doi:10.3390/ijms24108911)
Supplement: Supplementary file 1 [file ijms-24-08911-s001.zip › ijms-2404327-supplementary.pdf]

**Supplementary Table S1. List of primers used for quantitative real-time PCR.**

| Gene | Forward primer | Reverse primer | Species |
|---|---|---|---|
| ***53BP1*** | 5′-TCACTGCCATGGAGGAGC-3′ | 5′-GGATGCCTGGTACTGTTTGG-3′ | Mouse |
| ***Bax*** | 5′-ATCCAGGATCGAGCAGGGCG-3′ | 5′-ACTCGCTCAGCTTCTTGGTG-3′ | Mouse |
| ***Cyclin D1*** | 5′-TCCGCAAGCATGCACAGA-3′ | 5′-AGGGTGGGTTGGAAATGAACT-3′ | Mouse |
| ***Gadph*** | 5′-CATGGCCTTCCGTGTTCCTA-3′ | 5′-GCGGCACGTCAGATCCA-3′ | Mouse |

**Supplementary Table S2. Densitometry of bands shown in Western blots in Figure 4E and Figure 5A.**

| | **Cerebellum** | **Vehicle** | **DOX-free** | **DOX-TBSV-CooP** |
|---|---|---|---|---|
| **Bim** | 13 | 16432 | 24731 | 24628 |
| **Cyclin** | 9821 | 22140 | 22659 | 27553 |
| **Tubulin** | 27388 | 24394 | 24076 | 20956 |

| | **Cerebellum** | **MB1** | **MB2** | **MB3** |
|---|---|---|---|---|
| **H-FABP** | 2387 | 5487 | 5284 | 3284 |
| **Tubulin** | 18454 | 16976 | 16473 | 18764 |

Data have been obtained by iBright Analysis Software, Thermo Fisher Scientific, Waltham, MA, USA.

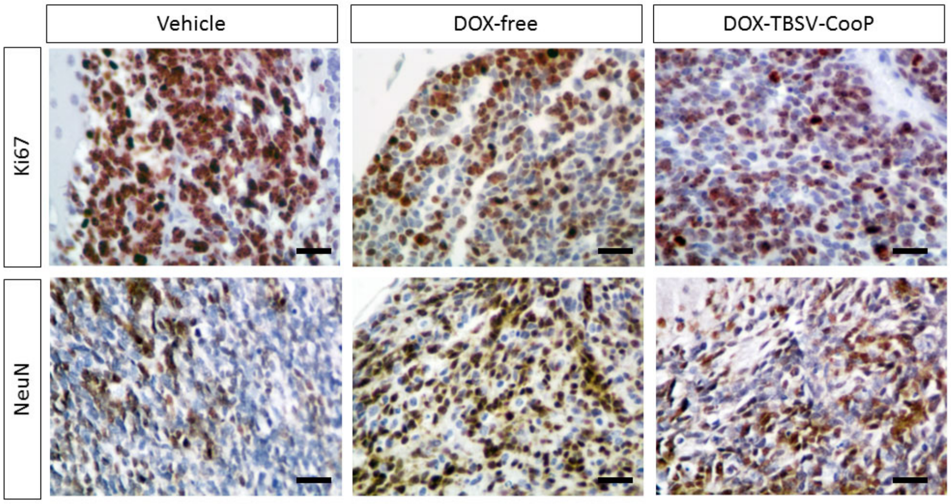


**Figure S1**. Immunostaining with Ki67 and NeuN antibodies. Representative histological images of MB PNLs from vehicle-, DOX-free and DOX-TBSV-CooP treated mice. Bars = 50 μm

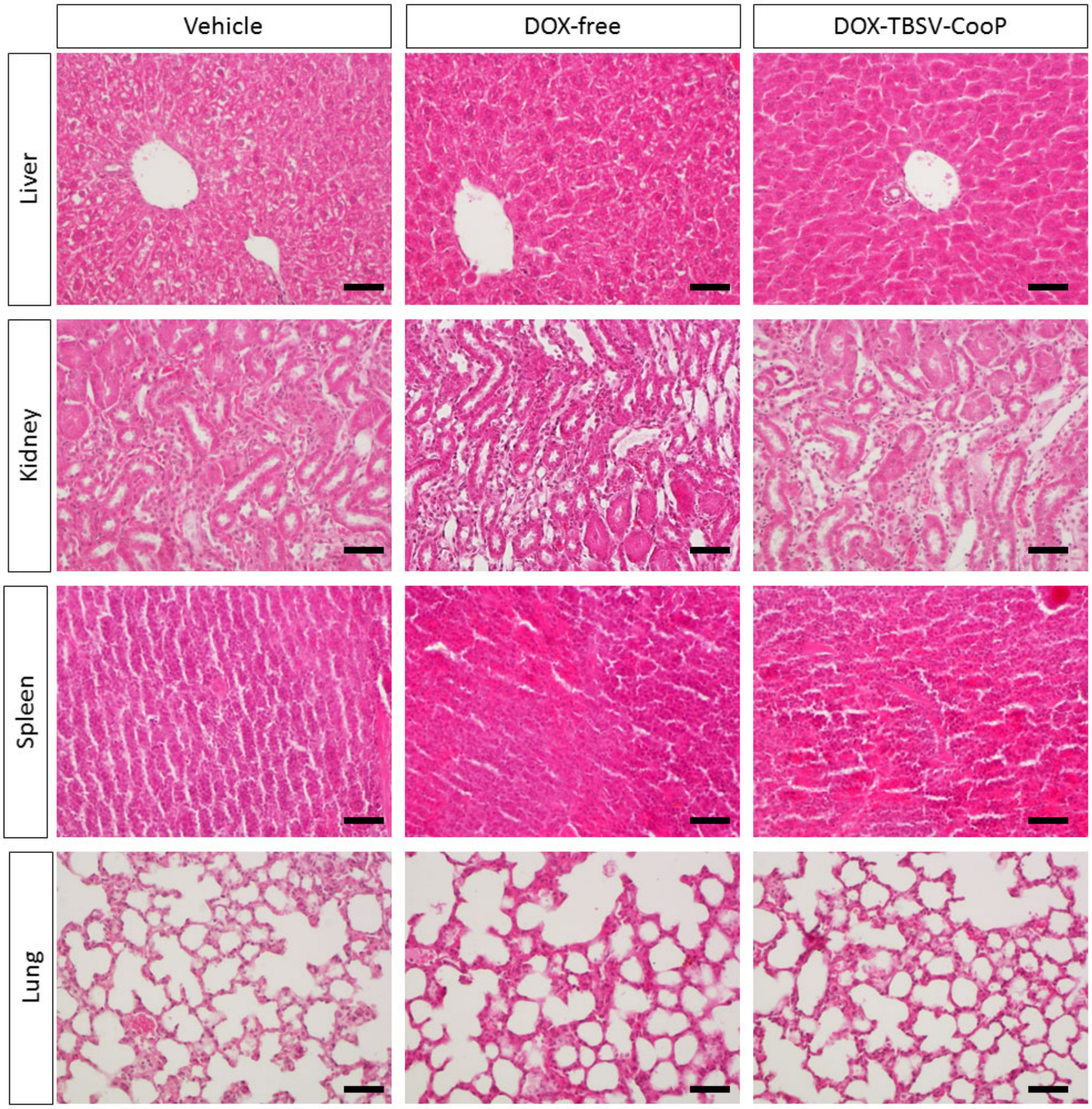


**Figure S2**. Microscopic examination of off-target organs. Representative histological images of organs collected from vehicle-, DOX-free and DOX-TBSV-CooP treated mice. Bars = 50 μm
